# Distribution of Porcine Cytomegalovirus in Infected Donor Pigs and in Baboon Recipients of Pig Heart Transplantation

**DOI:** 10.3390/v10020066

**Published:** 2018-02-06

**Authors:** Uwe Fiebig, Jan-Michael Abicht, Tanja Mayr, Matthias Längin, Andrea Bähr, Sonja Guethoff, Almuth Falkenau, Eckhard Wolf, Bruno Reichart, Tomoyuki Shibahara, Joachim Denner

**Affiliations:** 1 Department of HIV and other Retroviruses, Robert Koch Institute, 13535 Berlin, Germany; FiebigU@rki.de; 2 Department of Anaesthesiology, Ludwig-Maximilians-Universität München, 81377 Munich, Germany; Jan.Abicht@med.uni-muenchen.de (J.-M.A.); Tanja.Mayr@med.uni-muenchen.de (T.M.); Matthias.Laengin@med.uni-muenchen.de (M.L.); 3 Molecular Animal Breeding and Biotechnology, Gene Center, Ludwig-Maximilians-Universität München, 85764 Oberschleißheim, Germany; a.Baehr@gen.vetmed.uni-muenchen.de (A.B.); ewolf@lmb.uni-muenchen.de (E.W.); 4 Medizinische Klinik und Poliklinik, Klinikum Rechts der Isar, Technische Universität München, 81675 Munich, Germany; 5 Walter Brendel Centre of Experimental Medicine, Ludwig-Maximilians-Universität München, 81377 Munich, Germany; Guethoff@t-online.de (S.G.); Bruno.Reichart@med.uni-muenchen.de (B.R.); 6 Department of Cardiac Surgery, Ludwig-Maximilians-Universität München, 81377 Munich, Germany; 7 Institute of Veterinary Pathology at the Centre for Clinical Veterinary Medicine, Ludwig-Maximilians-Universität München, 81377 Munich, Germany; falkenau@patho.vetmed.uni-muenchen.de; 8 Pathology and Pathophysiology Research Division, National Institute of Animal Health, Tsukuba 305-0856, Japan; tshiba@affrc.go.jp; 9 Robert Koch Fellow, Robert Koch Institute, 13353 Berlin, Germany

**Keywords:** porcine cytomegalovirus, virus transmission, xenotransplantation, virus safety

## Abstract

The porcine cytomegalovirus (PCMV) is a herpesvirus that may pose a risk for xenotransplantation using pig cells, tissues, or organs. Here, three orthotopic pig heart transplantations into baboons were studied. To detect PCMV, a real-time PCR and a Western blot assay based on four PCMV protein sequences, including two tegument proteins, were used. The transmission of PCMV from the donor pig to the recipient baboon was found in two cases, despite PCMV not being detected in the blood of the donor pigs by real-time PCR. Although it was not in the blood, PCMV was detected in different organs of the donor pigs, and in sibling animals. Immunohistochemistry using an antiserum that is specific for PCMV detected virus protein-expressing cells in all of the organs of the recipient baboon, most likely representing disseminated pig cells. Therefore, for the first time, the distribution of PCMV in organs of the donor pigs and the recipient baboons was described. In addition, baboon cytomegalovirus (BaCMV) was found activated in the recipient, and a screening for hepatitis E virus (HEV) and porcine lymphotropic herpesviruses (PLHV) was performed. For the first time, a cross-reactivity between antibodies directed against PCMV and BaCMV was found.

## 1. Introduction

Xenotransplantation is at present the most promising solution for overcoming the increasing shortage of human organs for the treatment of organ failure [1]. This shortage is the reason why a large number of individuals die on the waiting list for the required organ [2]. Pigs are for several reasons the most suitable donor species. These reasons include the size of the organs, the functional compatibility (for example, pig insulin has been used successfully for the treatment of diabetes over decades), and the ability to create cloned and genetically modified pigs in a short time [3,4]. However, the transplantation of pig cells, tissues, and organs may not only save and prolong human life, it may also be associated with the transmission of potentially zoonotic porcine microorganisms, and the porcine cytomegalovirus (PCMV) among them [5,6]. PCMV is widely distributed among pigs; it is a rather stable DNA virus that can easily be transmitted from pig to pig by milk, urine, and faeces [7,8]. Transplacental transmission of PCMV has also been described [8,9]. When evaluating the risk posed by PCMV, it was found that the survival time of pig kidneys from PCMV-infected pigs transplanted into baboons [10] or cynomolgus monkeys [11] was significantly reduced, indicating that PCMV may also pose a risk for clinical xenotransplantation [12,13,14]. The transmission of PCMV was also observed after pig heart transplantation, which was associated with injury of the transplant, and an increased incidence of consumptive coagulopathy [15]. Early weaning excluded PCMV, resulted in a prolonged survival of the transplant, and prevented consumptive coagulopathy [15].

To detect PCMV in pigs, sensitive PCR-based methods [16,17,18,19,20] have been developed, as well as immunological methods screening for PCMV-specific antibodies as an indirect marker of virus infections [21,22,23]. Screening pigs for PCMV demonstrated that sensitive PCR-based methods have to be used, since less sensitive methods gave false-negative results [18]. To eliminate PCMV from pig herds, elimination programs based on treatment, early weaning, colostrum deprivation, Caesarean delivery, and embryo transfer have been proposed (for a review, see [24]).

Recently, we reported on the transmission of PCMV after transplantation of a pig heart into a baboon, even though PCMV was not detected in the blood of the donor pig [25]. Here, this and two other cases of orthotopic pig heart transplantations were analysed in detail, and for the first time, an analysis of the prevalence and tissue distribution of PCMV in donor pigs and the recipient baboons is reported.

## 2. Materials and Methods

### 2.1. Animals and Transplantations

All of the donor pigs (3701, 5154, 5292) and their siblings (5157, 5160 to 5154) were triple genetically modified; all were crossbreeds of German Landrace and Large White, with an alpha1,3-galactosyltransferase gene-knockout (GalT-KO), expressing human CD46 and human thrombomodulin (hTM) (Revivicor, Blacksburg, VA, USA, and Institute for Molecular Animal Breeding and Biotechnology, Faculty of Veterinary Medicine, LMU, Munich, Germany). GalT-KO pigs do not express the Gal epitope, which is the major cell surface determinant toward natural occurring antibodies that induce the so-called hyperacute rejection (HAR). Animal 5014 was a male Landrace pig with GalT-KO and expressing hTM, animal 5016 was a male Landrace pig with GalT-KO, and animal 5017 was a male Landrace pig with GalT-KO expressing CD46, all born 4 May 2016.

The first transplant recipient, a four-year-old male baboon (#57), was transplanted and treated as described [25]. The transplantation and treatment of baboons 64 (male, four years old) and baboon 62 (male, four years old) was performed similarly.

For the establishment of a new testing strategy, peripheral blood monoculear cells (PBMCs) from pigs 89030, 91106, 91107, 91111, 91112, 91117, 01118, 91119, and 91122 were used. These pigs were DanAvl basic hybrid sows; they are not genetically modified, and they were derived from a specified pathogen-free (SPF) facility in Fehmarn, Germany. In December 2016, at an age of approximately 11 months, they had been tested positive for hepatitis E virus (HEV) using PCR, and negative for porcine lymphotropic herpesviruses (PLHV)-1, -2, -3 and porcine circovirus 2 (PCV2) using PCR.

The baboons were obtained from the German Primate Center, Göttingen, Germany. Housing of the baboons was in accordance with national regulations (see Ethic statement). Enrichment for primates was individually adapted to each animal. In addition, a “clicker training” was performed. Blood samples were taken from the pigs and baboons after the induction of anesthesia before the transplantation. Second blood samples, as well as tissue samples, were taken after euthanisation of the baboons.

Premedication of the pigs consisted of intramuscular injections of ketamine hydrochloride 10–20 mg/kg (Ketavet^®^ 100 mg/mL, Pfizer Deutschland GmbH, Berlin, Germany), azaperone 10 mg/kg (Stresnil^®^ 40 mg/mL, Lilly Deutschland GmbH, Bad Homburg, Germany), and atropine sulfate (Atropinsulfat B. Braun 0.5 mg, B. Braun Melsungen AG, Melsungen, Germany). General anesthesia was induced with an intravenous bolus of 20 mg propofol (Propofol^®^-Lipuro 2%, B. Braun Melsungen AG, Melsungen, Germany) and 0.05 mg fentanyl (Fentanyl-Janssen 0.5 mg, Janssen-Cilag GmbH, Neuss, Germany). Anesthesia was maintained with the continuous infusion of propofol 7 mg/kg/h; analgesia was achieved by intravenous bolus injections of 2.5 µg/kg fentanyl repeated every 30 min if necessary. Donor pigs were euthanised by applying cardioplegia and subsequently explanting the heart in deep anesthesia.

Premedication of the baboons consisted of an intramuscular injection of ketamine hydrochloride 10 mg/kg and 0.5 mg/kg midazolam (Midazolam-ratiopharm, ratiopharm GmbH, Ulm, Germany). General anesthesia was induced with an intravenous bolus of 1.5–2.5 mg/kg propofol and 0.05 mg fentanyl. Anesthesia was maintained with the continuous infusion of 5–15 mg/kg propofol, and analgesia was achieved by intravenous bolus injections of 6–8 μg/kg fentanyl repeated every 45 min. A continuous infusion of fentanyl, ketamine hydrochloride, and metamizole (Novaminsulfon-ratiopharm^®^ 1 g/2 mL, ratiopharm GmbH, Ulm, Germany) was applied after transplantation to ensure analgesia. Baboons were euthanised by explanting the pig heart in deep anesthesia.

Orthotopic pig-to-baboon cardiac xenotransplantations were carried out according to the technique of Lower and Shumway [26]. Telemetric transmitters were implanted during surgery, which allowed for continuous post-surgical cardiovascular monitoring (blood pressure and electrocardiography). During the early postoperative period, a fixed regime of fentanyl, ketamine hydrochloride, and metamizole was administered. Analgesic treatment was then adjusted and stopped depending on clinical inspection (activity, posture, feed intake, contacting) and hemodynamic parameters (blood pressure, heart rate) as indirect signs of pain and distress. If necessary, analgesic treatment was restarted during the experiment.

Score sheets were used to describe the animals’ distress. Humane endpoints were defined by various conditions resistant to treatment such as automutilation, anorexia, vomitus, diarrhoea, impaired wound healing, bleeding, and pleural effusions. The experiment was terminated if one or more of these conditions were observed, or if a predefined maximum score was exceeded.

All animals were euthanised when humane endpoints were reached. No mortality occurred outside of planned euthanasia or humane endpoints.

### 2.2. Ethics Statement

Both the generation of transgenic animals, as well as interventions on re-cloned animals, were performed with permission of the local regulatory authority, Regierung von Oberbayern (ROB), Sachgebiet 54, 80534 München (approval numbers, AZ 55.2-1-54-2532-70-12, 20 November 2012 and AZ 55.2-1-54-2532-163-14). Applications were reviewed by the ethics committee according to §15 TSchG German Animal Welfare Law. The xenotransplantation experiment was approved by the Government of Upper Bavaria, Munich, Germany (reference number 55.2-1-54-2532-184-2014, September 2015). Housing, feeding, environmental enrichment, and steps taken to minimise suffering, including the use of anesthesia and method of sacrifice, was in accordance with the recommendations of the Weatherall report “The use of non-human primates in research”.

### 2.3. Testing for PCMV, HEV and BaCMV, PCR Methods

PCMV testing was performed as described [17,21,27,28] using specific primers (Table 1). Briefly, DNA was extracted from sera and blood of the pigs using two DNA extraction kits, a DNeasy Blood and Tissue kit (Qiagen GmbH, Hilden, Germany) and a ZR viral DNA kit (Zymo Research Corp., Irvine, CA, USA). DNA was quantified using a NanoDrop ND-1000 (Thermo Fisher Scientific Inc., Worcester, MA, USA). DNA was isolated from organs using the DNeasy Blood & Tissue Kit. To screen for PCMV, a real-time PCR with a detection limit of two to five copies was performed using a SensiFast probe no ROX one-step kit, according to supplier recommendations (Bioline GmbH, Germany). Then, 60 ng of DNA were used for testing. The reaction mixture contained 400 nM of both primers, and 100 nM of the probe (Table 1) in a final volume of 20 μL. The following conditions for amplification were used: denaturation at 95 °C for 5 min, and 45 cycles of amplification with denaturation at 95 °C for 10 s, annealing at 59 °C for 20 s, and extension at 60 °C for 25 s. Reporter fluorescence was measured using an Mx3005P Multiplex Quantitative PCR System (Stratagene, La Jolla, CA, USA).

To test for HEV, RNA was isolated and a real-time PCR was performed as described [29,30] using specific primers (Table 1).

To test for baboon cytomegalovirus (BaCMV), a real-time PCR was performed as described [31] using specific primers (Table 1).

### 2.4. Determination of the PCMV Copy Number

To determine the copy number of PCMV, a standard plasmid covering the target DNA of the PCMV PCR was generated. For this, the amplificate was cloned into a TOPO vector using the Zero Blunt TOPO PCR kit (Invitrogen Corporation, Carlsbad, CA, USA). The generated plasmid was diluted from 2 × 10^5^ to 0.1 copies per reaction to obtain a standard curve by real-time PCR. DNA from different organs, and the blood before and post mortem of baboon 64 and the donor pig, were analysed.

### 2.5. Determination of the BaCMV Copy Number

A droplet digital PCR was established to measure the BaCMV copy numbers using the QX200™Droplet Digital™PCR System (Bio-Rad Laboratories Inc., Hercules, CA, USA). PCR reactions were carried out as duplex PCR using baboon CCR5 as a housekeeping gene. Primer and probes were adopted from Mueller et al. [31]. PCR reaction mixes were prepared as follows: 10 µL of 2*ddPCR™ Supermix for Probes (No dUTP, Bio-Rad Laboratories Inc.), 180 pmol of each primer, and 50 pmol probe were mixed with 2 ng template DNA, isolated as described above. To complete the 20-µL reaction volume, the appropriate amount of nuclease and protease free water was added. BaCMV copy numbers were calculated using QuantaSoft (Bio-Rad, version 1.7.4.0917).

### 2.6. PBMC Incubation

Porcine PBMC were isolated by Ficoll gradient centrifugation (lymphocyte separation medium, PromoCell, Heidelberg, Germany), using Falcon tubes without porous barriers. The isolated PBMCs were washed with culture medium (RPMI1640, antibiotics, glutamine) and 10% foetal calf serum (FCS) (Merck Millipore, Darmstadt, Germany). DNA was isolated before and after incubation. In some experiments, PBMCs were incubated with mitogen phytohemagglutinin (PHA) (10 µg/mL, Millipore).

### 2.7. PCMV and HEV Antigens Used for Western Blot Analyses

In Western blot analyses for the detection of antibodies against PCMV, two His-tagged recombinant parts of the glycoprotein gB of PCMV, designated R1 (N-terminal part) and R2 (C-terminal part), were cloned, produced, purified, and used as described [21]. In addition, two newly produced tegument proteins of PCMV (U54A and U54B) [37] were cloned, produced, and purified as described [23]. Briefly, the U54A sequence was located from 70,307-72,304 (protein ID, AGT99246.1, GenBank No. KF017583), and the sequence of U54B was located from 72,345–73,541 (protein ID, AGT99247.1, GenBank No. KF017583). The sequences were codon optimised using Jcat, a novel tool to adapt codon usage [38] for bacterial expression, and synthesised by ATG biosynthetics (Merzhausen, Germany). The sequences were cloned into the expression vector pet16b with NdeI and XhoI restriction sites (Novagen, Madison, WI, USA). The cloned sequences were confirmed by DNA sequencing. Both of the sequences were expressed as recombinant His-tagged fusion proteins in *E. coli* BL21cells (New England Biolabs, Frankfurt/Main, Germany). The constructs were grown in 2 L 2YT-Medium at 37 °C until an optical density (OD) of 0.7, followed by induction with 1 M isopropyl β-d-1-thiogalactopyranoside (IPTG). After 3 h of induction, cells were pelleted by 8000 rpm for 15 min, and stored at −20 °C until purification. Cell pellets were resuspended in buffer (PBS, 1 mg/mL lysozyme, Sigma-Aldrich, St. Louis, MO, USA, and 50 µL benzonase, Merck Millipore, Darmstadt, Germany), sonicated three times for 20 s, and incubated on ice for 20 min. The cell debris was removed by centrifugation (11,000 rpm, 10 min), and pellets were extracted with lysis buffer (6 M guanidinium chloride, 500 mM NaCl, 20 mM disodium phosphate pH 7.5) overnight at room temperature. Solubilised proteins were separated from the remaining insoluble material by centrifugation (2 × 11,000 rpm, 15 min), diluted to 100 mL lysis buffer, and loaded on HisTrap 5-mL excel columns (GE Healthcare, Buckinghamshire, UK). The columns were equilibrated with lysis buffer, and loaded with solubilised proteins. After washing with lysis buffer and a second wash buffer (8 M urea, 500 mM NaCl, 30 mM (UA) or 20 mM (UB) imidazole, 20 mM disodium phosphate pH 7.5), the proteins were eluted using a 10-column volume gradient with elution buffer (8 M urea, 500 mM NaCl, 500 mM imidazole, 20 mM disodium phosphate pH 7.5).

In order to detect antibodies against HEV, the GT3 antigen was used [29,30].

### 2.8. Testing for PCMV, HEV and PLHV

Western blot analysis was performed using 500 ng/lane of the corresponding antigen. The proteins were dissolved in sample buffer (50 mM Tris-HCl, 12% glycerol, 4% SDS, 5% β-mercaptoethanol, 0.01% bromophenol blue), and denaturated for 5 min at 95 °C prior to electrophoresis. The proteins were analysed using 10% or 14% polyacrylamide gel and the PageRuler prestained protein ladder (ThermoFisher, Waltham, MA, USA). Proteins were transferred for 50 min to nitrocellulose membranes by electroblotting (15 V), stained with Ponceau red, cut into strips, and blocked overnight at 4 °C with 5% blotting grade dry milk (Roth) in PBS with 0.05% Tween 20 (blocking buffer). Strips were incubated with sera diluted 1:300 in blocking buffer for 2 h at room temperature. Polyclonal goat anti-pig immunoglobulin G (IgG)–alkaline phosphatase (AP) (Abcam, Cambridge, UK) was diluted 1:1000 in blocking buffer. For the detection of the gB protein, a 1:1000 dilution of the Penta-His antibody (Qiagen, Hilden, Germany) as the primary antibody, and 1:1000 polyclonal rabbit anti-mouse IgG–horseradish peroxidase (HRP) or IgG–AP (Dako, Hamburg, Germany) were used as well. Staining was performed with 3,3′-diaminobenzidine (DAB) (Thermo Fisher) or with 5-bromo-4-chloro-3- indolyl-phosphate nitro blue tetrazolium (NBT/BCI) (Promega, Madison, WI, USA). Testing for PLHV-1, -2, and -3, using PCR assays and Western blot analyses, were performed as described [39].

### 2.9. Immunohistochemistry (IHC)

A PCMV-specific rabbit polyclonal antibody was used in this study, which was produced by immunising a rabbit with PCMV J1, which was the first field isolate in Japan. Immunohistochemistry (IHC) was performed as described previously [40] using the universal immune-enzyme polymer method with a Histofine Simple Stain MAX-PO Kit (Nichirei, Tokyo, Japan). The serial sections were pretreated with 0.1% actinase for 20 min at 37 °C, and endogenous peroxidase activity was blocked using 3% H_2_O_2_ in methanol for 30 min at room temperature. The obtained antibody was applied as the primary antibody at a dilution of 1:1024 for 30 min at room temperature. The sections were lightly counterstained with hematoxylin, and assessed using light microscopy. Simultaneously, sections of porcine kidney that were known to be infected with PCMV were immunolabelled as positive controls. Negative controls were prepared by replacing the primary antibody with a commercial Tris-HCl buffer (antibody diluent with background reducing components) (Dako, Tokyo, Japan).

### 2.10. Sequence Alignment

In order to confirm the immunological cross-reactivity between PCMV and BaCMV at the sequence level, an alignment of the sequences of the glycoproteins of both viruses was performed using BioEdit version 7.2.5, pairwise alignment (Ibis Biosciences, Carlsbad, CA, USA) [41].

## 3. Results

### 3.1. Time Schedule of the Transplantation Experiments and Testing for PCMV

**Figure 1:**
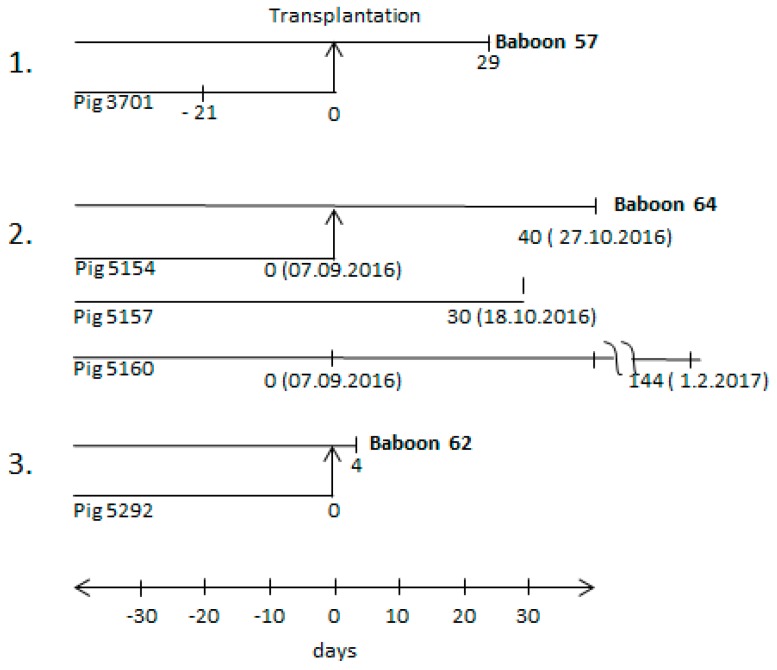
Time schedules of three different transplantation experiments (numbers 1–3) and animals involved. The arrows from the line of the donor pig (3701, 5154, 5292) to the line of the recipient baboon (57, 64, 62) indicate the day of transplantation (day 0); other time points before and after transplantation indicate the time of bleeding. The bleeding times of sibling pigs 5157 and 5169 are also indicated.

Three orthotopic pig heart transplantations into baboons were performed (Figure 1). The donor pigs (pig 3701, pig 5154, and pig 5292) were all genetically modified, as described in the materials and methods section. The first transplantation experiment has been partially described [25,30]; the two others were performed similarly with slight modifications. In addition, two sibling pigs to donor pig 5154, pig 5157, and pig 5160 were also analysed.

### 3.2. Detection of PCMV in Donor Pig and Baboon Recipients

All three donor pigs (3701, 5154, and 5292) were found negative when the blood of these animals was analysed by PCR for PCMV infection. Although PCMV was not detected by real-time PCR in the blood of donor pig 3701, the virus was transmitted to the recipient baboon 57 and a high virus titre in the recipient suggested replication in the transplant and/or recipient [25]. Whereas the testing of animal 3701 had been performed 21 days before transplantation (Figure 1), in the second transplantation experiment, the blood from donor pig 5154 was tested directly on the day of transplantation (day 0). In parallel, animal 5154 and two sibling animals tested positive for hepatitis E virus (HEV) and negative for porcine lymphotropic herpesviruses (PLHV-1, PLHV-2, PLHV-3) (Table 2). However, when animal 5160 and sibling 5157 were tested several weeks after the transplantation of the heart from donor animal 5154, blood from both of the pigs was found positive for PCMV. To note, the blood of animal 5154 was negative for PCMV on the day of transplantation (day 0). The fact that PCMV was detected in the blood of sibling animals 5160 and 5157 on day 30 and day 144 suggests that PCMV was replicating. It is likely that the whole litter had been infected by the mother, since early weaning was not performed. However, the virus did not induce antibodies against the glycoprotein (gB) of PCMV, as shown by Western blot results, using the N-terminal part R1 and the C-terminal part R2 of gB as antigen (Table 2).

The recipient of the heart from pig 5154, baboon 64, was found PCMV-positive 40 days after transplantation (see below). In the third transplantation experiment (Figure 1), blood from donor pig 5292 was also found PCMV-negative on the day of transplantation. In this case, the recipient baboon 62 deceased four days later, and it was found PCMV-negative, certainly due to the short survival time of the transplant.

Although the donor pigs were HEV-positive as shown by real-time PCR, baboons 57 and 64 were HEV-negative [30,42].

### 3.3. Distribution of PCMV in Pig Organs

**Figure 2:**
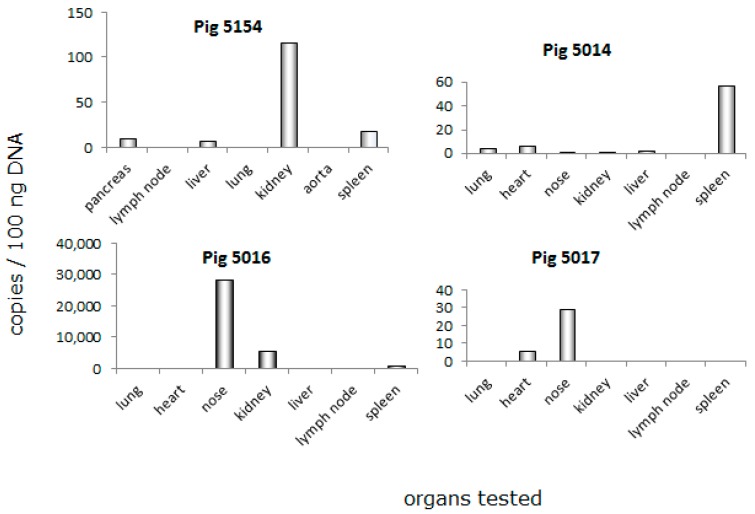
Real-time PCR analysis of PCMV distribution in different organs of donor animal 5154, and other animals (5014, 5016, and 5017). DNA was isolated and tested for the copy number of PCMV. Please note the differences in scaling of the *y*-axis.

When the blood of donor pig 5154 was tested, it was found negative for PCMV on the day of transplantation (day 0, Figure 1). To identify the reservoir of PCMV leading to the infection of baboon 64, different organs of this animal were screened using a real-time PCR. PCMV was found mainly in the kidney, but also in the pancreas, liver, and spleen of animal 5154; in other organs of this animal (lymph node, lung, and aorta), the virus was under the detection limit of the method (Figure 2). When in parallel, the organs from three related and also genetically modified pigs—5014, 5016 and 5017—were tested for PCMV by real-time PCR (Figure 2), in these animals, the virus was found in all of the organs analysed. The highest virus load was found in the nose (pigs 5016, 5017), the spleen (pig 5014), and lower amounts were found in the heart (pig 5017) and kidney (pig 5016) (Figure 2). These data indicate that (i) the copy number differed considerably from animal to animal (compare the virus load in the nose of pig 5016 with that in the nose of pig 5017); and (ii), with exception of the nose, in each animal, another organ showed the highest copy number (Figure 2). These differences were not based on different ages or different litters, since animals 5014, 5016, and 5017 were born on the same day from the same mother.

In addition, tissue samples from pigs 5014 and pig 5016 were analysed by immunohistochemistry (IHC), using antibodies specific for PCMV. No PCMV was detected using this method in the liver, spleen, kidney, heart, lung, and lymph node of both animals [43]. When tissue samples from the nose (nasal mucosa and conchen) of animal 5016 were investigated, no PCMV could be detected likewise. The difference in the PCR results showing the presence of the virus in some organs (Figure 2) is certainly due to the lower sensitivity of the IHC method, or due to some cells containing viral DNA, but not expressing the protein.

### 3.4. Development of a New Detection Strategy Using Incubated PBMC

**Figure 3:**
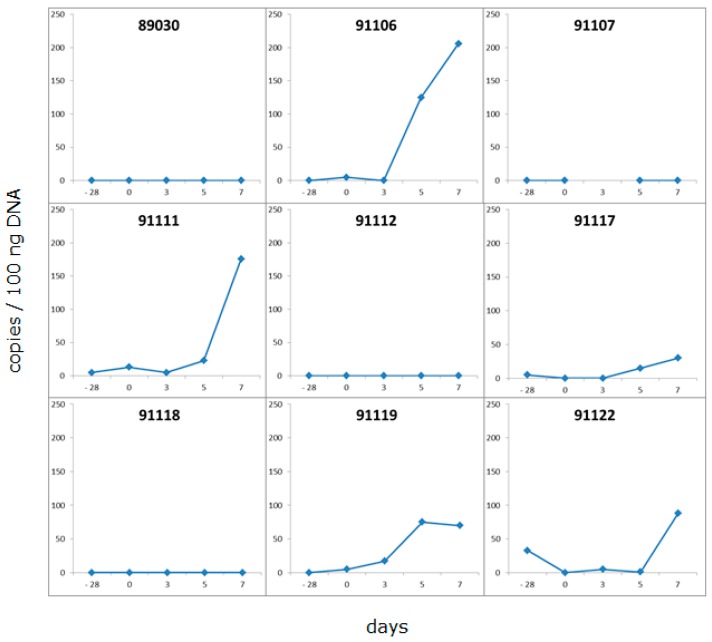
Real-time PCR analysis of PBMCs from PCMV-infected pigs before and after incubation in culture medium. Ficoll gradient-isolated PBMCs were incubated in culture medium for five days; DNA was isolated before and after incubation, and tested for PCMV. The value 0 at day −28 indicates the absence of PCMV in blood samples taken 28 days before the incubation experiment.

Since pig 5154 was PCMV-positive in a few organs, but negative when the blood was tested using the same real-time PCR, an analysis was performed as to whether testing for PCMV in the blood can be improved by incubating the isolated PBMCs from the animal under study for five to seven days in culture medium. Freshly isolated PBMCs from nine pigs were incubated and tested before and after incubation (Figure 3). These animals were DanAvl basic hybrid sows; they were not genetically modified, and they had been derived from a SPF facility in Fehmarn, Germany. Twenty-eight days before this experiment, blood samples from these animals tested negative for PCMV using PCR. After cultivation of the pig PBMCs, a significant increase in the amount of detected virus was observed in two of nine cases (pigs 91106, 91111), and a moderate increase was observed in three of nine cases. In four cases, no virus was detected; presumably, these animals were truly negative, at least in the blood samples and using our detection method. Stimulating the PBMCs with the mitogen PHA did not increase the virus replication. These data indicate that PCMV was replicating during cultivation, and that this approach increases the efficacy of virus screening in the blood, which may be a great advantage when testing pigs. This is a very important finding. Without incubation, all nine animals would have been declared PCMV-negative, but using this assay, five animals were found PCMV-positive.

### 3.5. Distribution of PCMV in Baboon Organs

**Figure 4:**
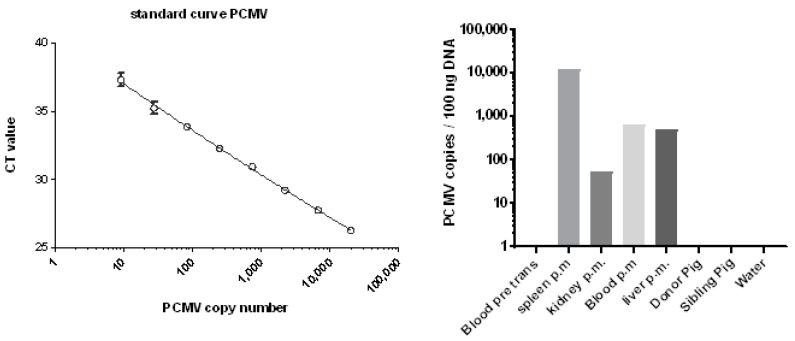
Standard curve for the estimation of the copy number of PCMV and copy numbers of PCMV in different organs of baboon 64 after orthotopic heart transplantation. DNA was isolated from different organs and tested using real-time PCR. Blood pre trans, blood before transplantation; p.m., post mortem; sibling pig is pig 5157; note the logarithmic scale of the axis.

Whereas in the case of baboon 57, no tissues with the exception of blood and serum were available to test for PCMV by PCR, in the case of baboons 62 and 64, different organs were available, and were tested using real-time PCR (Figure 4). In the case of baboon 64, high numbers of viral DNA were found in the spleen, and lower numbers of PCMV DNA copies were found in the blood and liver, whereas only very few copies were found in the kidney. Whereas the baboon blood was negative before transplantation, the blood after transplantation showed a detectable virus load (Figure 4). Material from the transplanted pig heart was not available for PCR testing, but was available for IHC studies (see below). In the case of baboon 62, all of the organs were negative [42], possibly due to the short survival time of the recipient.

**Figure 5:**
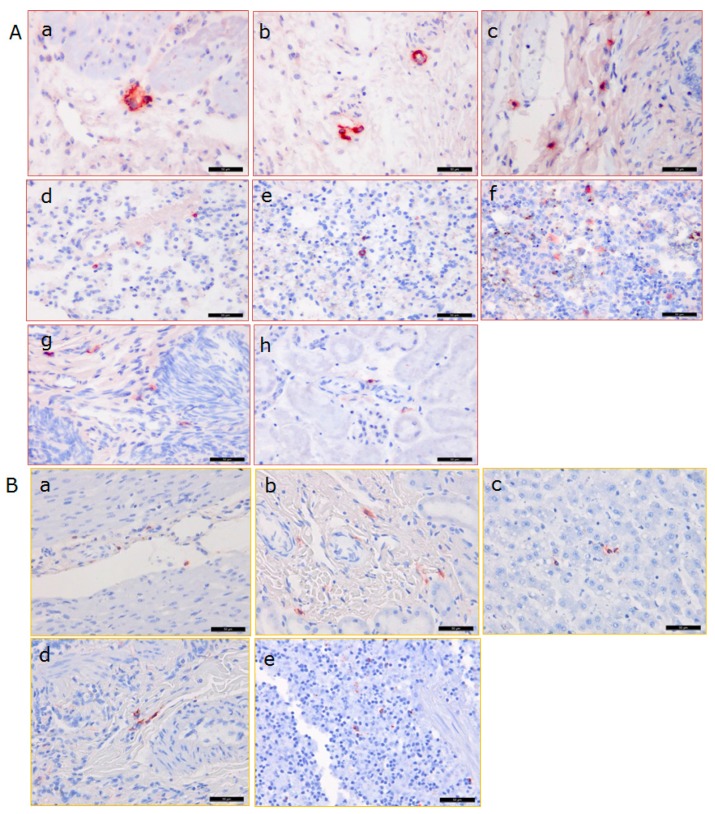
Immunohistochemical analysis of PCMV distribution in the pig donor heart, in different organs from baboon 57 (**A**) and from control baboon 43 (**B**). To detect PCMV, a specific rabbit serum was used, A, baboon 57, a,b—pig heart, c—baboon liver, d—baboon lung, e—baboon spleen, f—baboon lymph node, g—baboon testis, h—baboon kidney; B, control baboon 43, a—heart, b—kidney, c—liver, d—lung, e—spleen. Scale bar = 50 µm.

In order to confirm the PCR results and obtain additional information about the distribution of PCMV in the baboon, an IHC analysis was performed using PCMV-specific rabbit antibodies (Figure 5A).

**Figure 6:**
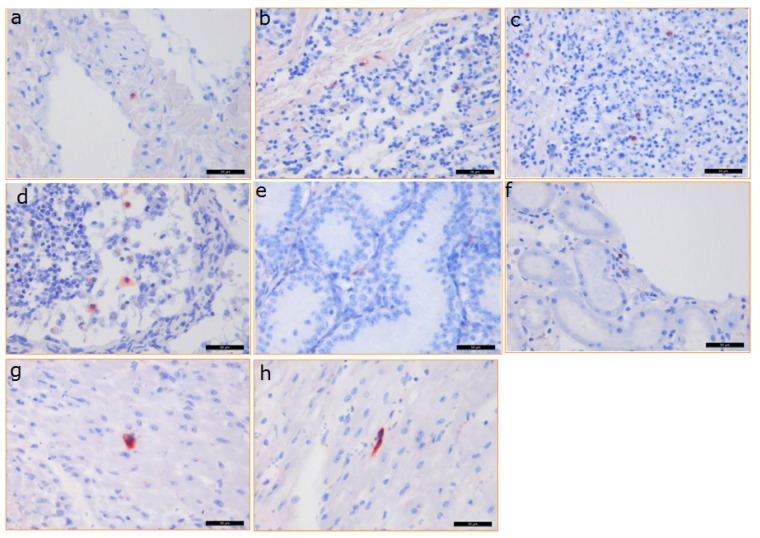
Immunohistochemical analysis of PCMV distribution in different organs of baboon 64. The same PCMV-specific rabbit serum was used as in Figure 5. a—baboon liver, b—baboon lung, c—baboon spleen, d—baboon lymph node, e—baboon testis, f—baboon kidney, g,h—pig heart. Scale bar = 50 µm.

In the case of baboon 57 (where unfortunately no organs were available to screen for PCMV by PCR), positive cells were found by IHC in the pig donor heart, as well as in all of the analysed organs of the baboon (liver, spleen, kidney, lung, lymph node, and testis) at the end of the experiment. The highest amount of positive cells was found in the baboon lymph node and testis, while a lower amount was found in the pig heart, and lower amounts were also found in the baboon liver, spleen, kidney, and lung. A semiquantitative presentation of the distribution in different organs as detected by IHC is given in Table 3. The same analysis was performed with tissues from baboon 64 (Figure 6, Table 3). A high number of PCMV-positive cells was detected in the lymph node and the spleen, correlating with the PCR result (Figure 4), as well as in the pig donor heart; a lower number was found in the liver, kidney, lung, and testis (Figure 6, Table 3).

### 3.6. Detection of the Baboon Cytomegalovirus (BaCMV) in the Recipient Baboons

**Figure 7:**
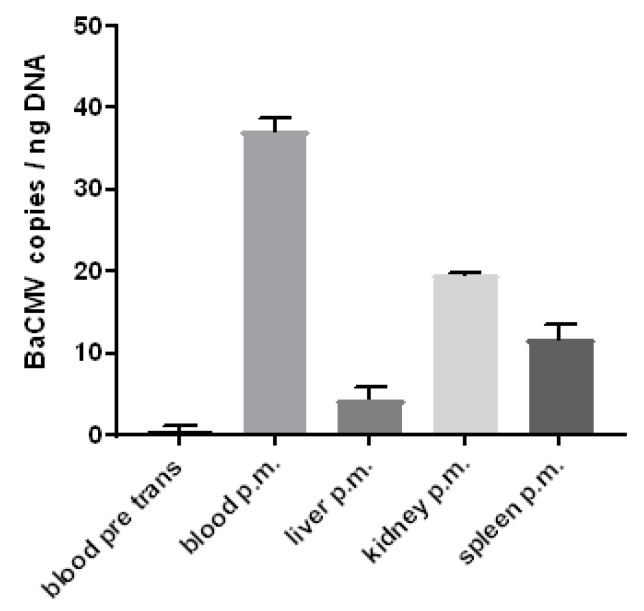
Prevalence of BaCMV in different organs of baboon 64. The comparison of the copy numbers of the virus in the blood before transplantation (blood pre trans) and after transplantation (post mortem, p.m.) clearly demonstrates the replication (activation) of BaCMV. In addition, the copy numbers in different organs at the end of the experiment were determined. For understandable reasons, the copy numbers in the organs before transplantation were not available.

Surprisingly, when a control baboon (number 43) was analysed by IHC using the rabbit serum against PCMV, a slightly positive reaction was observed in some cells in different organs (Figure 5B, Table 3). Since this animal had no contact to pigs and an infection with PCMV thus can be excluded, several baboons were screened for BaCMV using a BaCMV-specific PCR method. This PCR does not detect PCMV. BaCMV DNA was found in all of the baboons tested so far; the distribution of BaCMV in the different organs of baboon 64 is shown in Figure 7. This animal was in parallel infected by PCMV, as shown by a PCMV-specific PCR (Figure 4). In contrast to the high load of BaCMV in the blood and different organs of baboon 64 at day 40 after transplantation, only a very low virus load was detected in the blood of baboon 64 before transplantation (Figure 7). The high virus load of BaCMV in the blood of animal 64 after the transplantation (Figure 7) indicated replication (activation) of the virus during the 40 days of transplantation, which was possibly influenced by the immunosuppressive regimen, or by ischemia reperfusion injury concomitant with release of cytokines, chemokines, and other factors. When control baboon 43 was tested by PCR for BaCMV, no virus was detected in the blood or serum, which was certainly due to the low virus load under the detection limit of our method. No other organs were available for PCR testing. BaCMV was also detected in the blood of baboon 57 at the end of the transplantation [42], suggesting that also in this case, the virus load increased during transplantation.

### 3.7. Immunological Cross-Reactivity between PCMV and BaCMV

Recently, a Western blot analysis was developed using two recombinant proteins corresponding to the N-terminal (R1) and the C-terminal (R2) parts of the glycoprotein B (gB) of PCMV, which had been used when screening for antibodies against PCMV in infected pigs [20]. Later, this Western blot analysis was extended using two PCMV tegument proteins, U54A and U54B, which had been cloned, expressed, purified, and used as antigens [22]. When all four antigens were used in order to analyse whether baboon 64 had developed antibodies against PCMV, a positive reaction was observed with the R2 protein and the U54A protein (Supplementary Figure S1). However, since no differences in the reactivity were observed between the serum before and after the transplantation (Supplementary Figure S1), it was suggested that this was not an antibody response against PCMV, but a cross-reaction against BaCMV.

To summarise, using two real-time PCR that specifically detect BaCMV or PCMV, both viruses were detected in baboons 57 and 64. When the sera of baboon 64 before and after the transplantation were analysed using Western blots with PCMV glycoprotein B and tegument proteins, a reactivity of the baboon sera with the PCMV proteins was observed, suggesting a immunological cross-reactivity (Supplementary Figure S1). Although only a few BaCMV sequences had been published, an alignment of the R2 region of the glycoproteins of PCMV and BaCMV confirmed the sequence homology in certain sequences (Supplementary Figure S2).

## 4. Discussion

Here, for the first time, the distribution of PCMV in the organs of genetically modified donor pigs used for orthotopic heart xenotransplantation and in organs of the transplanted baboons are described. It is important to note that PCMV was found in several organs of the donor pig—but not in the blood—when tested at an earlier time point, indicating that testing blood is not an efficient way to detect PCMV in young pigs. Based on this result, the testing of organs from sibling animals of the same litter is recommended. In addition, in a recent study, which was not yet finished when we analysed the pigs in these transplantation experiments, we observed that testing non-invasive oral and anal swabs by PCR may be more efficient for PCMV detection compared with testing blood [44]. However, incorrect sampling of the swabs may give wrong results. Therefore, in order to increase the efficacy of PCMV detection, pig PBMCs were cultivated and tested after five days. This strategy significantly increased the efficacy or testing, and did not have the uncertainties of swab collection. When the organs of the pigs were tested, the highest amount of PCMV was detected in the nose, followed by the kidney and spleen (Figure 2). These results correlate with previous publications: in one of these investigations, 38 PCMV genome copies/µg of DNA were found in the kidney, and 97 genome copies/µg of DNA were found in the lung [16]. The viral load in the liver, gut, and salivary gland were below the threshold of quantification. In general, the primary site of virus replication in pigs is believed to be in the nasal mucous glands, the lachrymal glands, or the Harderian glands. These organs were not available for testing in our study. The destruction of the cells in these organs results in marked rhinitis and conjunctivitis (this explains why PCMV infection had been previously designated inclusion body rhinitis). Widespread damage caused by the virus or secondary infections can lead to death [45]. It was shown that the replication of the latent PCMV can be significantly enhanced in transplanted organs [31] and in culture through allogenic stimulation [46].

As mentioned above, our results showing an increase in virus load after the in vitro cultivation of isolated pig PBMCs from infected animals (Figure 3) propose a more efficient testing for PCMV when only the blood of the animals is available. These results are in line with the re-activation of PCMV through allogenic stimulation, as has been shown earlier [46]. Although in our approach allogenic cells were not added, the cultivation alone or in vitro responses to proteins present in the foetal calf serum (FCS) of the culture medium may have activated PCMV. Although this method may significantly increase the sensitivity of PCMV detection, since it needs at least five days for cultivation, it is not suited for quick testing.

The detection of PCMV in cells in all of the organs of baboon recipients 57 and 64, as shown by IHC, is one of the most impressive results of this study. Although it is still unclear whether PCMV is able to infect baboon or human cells, most data indicate that herpes viruses are species-specific [47]. This was also shown, for example, for mouse cytomegalovirus (CMV) [48]. In the case of PCMV and human cells, one publication reported the infection of human fibroblasts [49], while another reported the failure of infection of human cells, showing that the co-cultivation of PCMV-infected pig macrophages with two human cell lines (293 and Raji) did not facilitate virus transmission [13]. Therefore, the question of whether PCMV can infect human cells and replicate in humans is still unanswered. Our data here suggest that porcine cells expressing PCMV antigens are found disseminated in the organism of the recipient baboon. Inclusion bodies, a characteristic of infection, were not detected in the positive cells; however, inclusion bodies were also occasionally not observed in previous investigations of infected animals [40]. Quantifying the amount of positive cells in different organs of baboon 57 using IHC analysis showed a very high amount of positive cells in the baboon lymph node and testis, a lower amount in the pig heart, and a low amount in the other tested baboon organs (liver, spleen, kidney, and lung) (Table 3). Similar results were obtained by IHC analysis for baboon 64. Interestingly, in baboon 64, an extremely high amount of PCMV DNA was detected in the spleen using real-time PCR. It remains unclear whether the difference in the findings obtained by PCR and IHC was due to the presence of viral DNA not expressing protein, or whether different parts of the organ with different expression profiles were used for PCR or IHC. Nevertheless, the highest amount of PCMV DNA was found in the spleen, while lower amounts were found in the blood and liver, and nearly no PCMV was found in the kidney (Figure 4).

Baboons are naturally infected with a cytomegalovirus, BaCMV, which has been isolated and described by several laboratories [50]. Most of the animals tested in baboon colonies are infected and have antibodies to BaCMV [51]. The immunological cross-reactivity between sera against PCMV and BaCMV is of interest, especially as PCMV is more closely related to HHV-6 and HHV-7 in comparison with HHV-5/HCMV. IHC analyses demonstrated that a rabbit serum against PCMV cross-reacts with BaCMV-infected cells in a baboon (Figure 5B), and a Western blot analysis showed that the serum from baboon 64 reacts with PCMV proteins before the pig heart transplantation and infection with PCMV. Recently, it has been reported that BaCMV was transmitted after a baboon-to-human liver xenotransplantion [52]. Replication-competent BaCMV was isolated from serial blood samples of the human recipient of the baboon liver on days 29, 36, and 42 after xenotransplantation. This was the first report of detecting a replication-competent virus from a source animal after xenotransplantation into a human, and was a concern with regard to potential zoonotic transmission to others [52]. Although it is still unclear whether PCMV infects human cells (see above), BaCMV replicates well in human cells in culture [53]. Human fibroblasts were permissive for BaCMV, isolates exhibited cytopathology similar to that of HCMV, and virions were observed by electron microscopy [53].

Although the severe shortage of human organs for transplantation is the driving force behind xenotransplantation research, non-human primates, particularly baboons, are for several reasons not potential sources of organs and tissues. One of these reasons is that BaCMV is endemic in baboon populations and infects human cells, and is therefore a potential cause of donor-associated disease after xenotransplantation. In addition, it is well known that the human cytomegalovirus (HCMV) is the most common donor-associated infection, and poses a major risk for allotransplantation. Therefore, all efforts should be undertaken to prevent the transmission of PCMV during xenotransplantation using cells, tissues, or organs from pigs.

## Figures and Tables

**Table 1:** Primers and probes used for real-time PCR. BaCMV: baboon cytomegalovirus; PLHV: porcine lymphotropic herpesviruses; PCMV: porcine cytomegalovirus

Primers Used for PCR	Sequence 5′-3′	Nucleotide Position	Accession Number	Reference
PLHV-1,-2 (747) fw PLHV-1,-2 (747) rev	CAYGGTAGTATTTATTCAGACA GATATCCTGGTACATTGGAAAG	21,146–21,167 21,488–21,467	AY170317.1	Ehlers B., 2002 [32]
PLHV-3 (905) fw PLHV-3 (905) rev	ACAAGAGCCTTAGGGTTCCAAACT GTGTCCAGTGTTGTAATGGATGCC	13,472–13,495 13,727–13,704	AY170316.1	Chmielewicz et al., 2003 [33]
Primers and probes used for real-time PCR				
pcyclophilin real fw pcyclophilin real rev pcyclophilin probe	TGCTTTCACAGAATAATTCCAGGATTTA GACTTGCCACCAGTGCCATTA Cy5-TGCCAGGGTGGTGACTTCACACGCC-BHQ	131–158 187–207 161–185	AY008846	Duvigneau et al., 2005 [34]
pGAPDH fw pGAPDH rev pGAPDH probe	ACATGGCCTCCAAGGAGTAAGA GATCGAGTTGGGGCTGTGACT HEX-CCACCAACCCCAGCAAGAG-BHQ1	1040–1062 1188–1168 1114–1132	NM_001206359.1	Duvigneau et al., 2005 [34]
JVHEVF JVHEVR JVHEVProbe	GGTGGTTTCTGGGGTGAC TGATTCTCAGCCCTTCGC FAM-TGATTCTCAGCCCTTCGC-BHQ	5283–5300 5352–5335 5306–5323	KT633715.1	Jothikumar et al., 2006 [35]
PCMV real fw PCMV real rev PCMV probe	ACGAGAAAGATATTCTGACGGTGCA TCTAGACGAAAGGACATTGTTGATA 6FAM-CAGGGCGGCGGTCGAGCTC-TAMRA	45,962–45,938 45,622–45,646 45,246–45,229	KF017583.1	Mueller et al., 2004 [36]
BaCMV real fw BaCMV real rev BaCMV probe	5′ GTTTAGGGAACCGCCATTCTG 3′ 5′ GTATCCGCGTTCCAATGCA 3′ 5′ 6FAM-TCCAGCCTCCATAGCCGGGAAGG-BBQ 3′	64,375–64,396 64,464–64,483 64,437–64,460	KR351281	Mueller et al., 2002 [31]
BaCCR5 real fw BaCCR5 real rev BaCCR5 probe	5′ TACCTGCTCAACCTGGCCAT 3′ 5′ TTCCAAAGTCCACTGGGC 3′ HEX-TTTCCTTCTTACTGTCCCCTTCTGGGCTC-BHQ 3′			Mueller et al., 2002 [31]

**Table 2:** Screening of animals 5154 (donor pig), 5160, and 5157 (sibling pigs) for potentially zoonotic porcine viruses. HEV: hepatitis E virus; PLHV: porcine lymphotropic herpesviruses.

Pig Number	Born	Gender	Material Collected (Date)	PCMV (Real-Time PCR)	HEV (Real-Time RT PCR)	PLHV1/2 (PCR)	PLHV3 (PCR)	PCMV (Western Blot) *	PLHV (Western Blot) **	HEV (Western Blot) ***
5154	1 July 2016	male	7 September 2016	no ct	no ct	no ct	no ct	negative	negative	negative
5160	1 July 2016	female	7 September 2016	no ct	no ct	no ct	no ct	negative	negative	negative
5160			3 February 2017	no ct	no ct	no ct	no ct	negative	negative	negative
5157	1 July 2016	female	26 October 2016	no ct	no ct	no ct	no ct	negative	negative	negative

* Using two antigens, R1 and R2, ** using one antigen, GB1, *** using one antigen, GT3, no ct–no threshold cycle value.

**Table 3:** Results of the immunohistochemistry (IHC) analysis of different organs from two baboons after pig heart transplantation and one control baboon.

Organ	Baboons		
	57	64	Control 43
Liver	+	+	+ *
Spleen	+	++	+ *
Kidney	+	+	+ *
Pig heart	++	++	+ *
Lung	+	+	+ *
Testis	+++	+	n.t.
Lymph node	+++	+++	n.t.

+ a few, ++ some, +++ many; * very weak reaction, cross-reactivity with BaCMV, n.t., not tested.

## Supplementary Materials

The following are available online at http://www.mdpi.com/1999-4915/10/2/66/s1. Figure S1: Western blot analyses using four antigens from PCMV and sera from baboon 64. M, marker, R1, recombinant fragment R1 of gB of PCMV, R2, recombinant fragment R2 of gB of PCMV, UA, tegument protein U54A, UB, tegument protein U54B, A, serum from baboon 64 before transplantation, B, serum from baboon 64 after transplantation, C, anti-His antibodies detecting the His tagged proteins, unfortunately UA was cleaved during production and purification, 1:1000, anti-mouse antibodies 1:1000. Figure S2: Alignment of the R1 and R2 regions of gB PCMV and BaCMV.

## References

[1] Ekser B., Ezzelarab M., Hara H., van der Windt D.J., Wijkstrom M., Bottino R., Trucco M., Cooper D.K. (2012). Clinical xenotransplantation, the next medical revolution?. Lancet.

[2] Tacke F., Kroy D.C., Barreiros A.P., Neumann U.P. (2016). Liver transplantation in Germany. Liver Transplant..

[3] Niemann H., Petersen B. (2016). The production of multi-transgenic pigs, update and perspectives for xenotransplantation. Transgen. Res..

[4] Klymiuk N., Aigner B., Brem G., Wolf E. (2010). Genetic modification of pigs as organ donors for xenotransplantation. Mol. Reprod. Dev..

[5] Fishman J.A., Patience C. (2004). Xenotransplantation, infectious risk revisited. Am. J. Transplant..

[6] Fishman J.A., Scobie L., Takeuchi Y. (2012). Xenotransplantation-associated infectious risk, a WHO consultation. Xenotransplantation.

[7] Edington N. (1986). Porcine cytomegalovirus. Dis. Swine.

[8] Edington N., Broad S., Wrathall A.E., Done J.T. (1988). Superinfection with porcine cytomegalovirus initiate infection. Vet. Microbiol..

[9] Mueller N.J., Kuwaki K., Knosalla C., Dor F.J., Gollackner B., Wilkinson R.A., Arn S., Sachs D.H., Cooper D.K., Fishman J.A. (2005). Early weaning of piglets fails to exclude porcine lymphotropic herpesvirus. Xenotransplantation.

[10] Yamada K., Tasaki M., Sekijima M., Wilkinson R.A., Villani V., Moran S.G., Cormack T.A., Hanekamp I.M., Hawley R.J., Arn J.S. (2014). Porcine cytomegalovirus infection is associated with early rejection of kidney grafts in a pig to baboon xenotransplantation model. Transplantation.

[11] Sekijima M., Waki S., Sahara H., Tasaki M., Wilkinson R.A., Villani V., Shimatsu Y., Nakano K., Matsunari H., Nagashima H. (2014). Results of life-supporting galactosyltransferase knockout kidneys in cynomolgus monkeys using two different sources of galactosyltransferase knockout swine. Transplantation.

[12] Clark D.A., Fryer J.F.L., Tucker A.W., McArdle P.D., Hughes A.E., Emery V.C., Griffiths P.D. (2003). Porcine cytomegalovirus in pigs being bred for xenograft organs, progress towards control. Xenotransplantation.

[13] Tucker A.W., Galbraith D., McEwan P., Onions D. (1999). Evaluation of porcine cytomegalovirus as a potential zoonotic agent in xenotransplantation. Transplant. Proc..

[14] Denner J., Mueller N.J. (2015). Preventing transfer of infectious agents. Int. J. Surg..

[15] Mueller N.J., Kuwaki K., Dor F.J., Knosalla C., Gollackner B., Wilkinson R.A., Sachs D.H., Cooper D.K., Fishman J.A. (2004). Reduction of consumptive coagulopathy using porcine cytomegalovirus-free cardiac porcine grafts in pig-to-primate xenotransplantation. Transplantation.

[16] Goltz M., Widen F., Banks M., Belak S., Ehlers B. (2000). Characterization of the DNA polymerase loci of porcine cytomegalovirus from diverse geographical origins. Virus Genes.

[17] Fryer J.F.L., Griffiths P.D., Fishman J.A., Emery V.C., Clark D.A. (2001). Quantitation of porcine cytomegalovirus in pig tissues by PCR. J. Clin. Microbiol..

[18] Morozov V.A., Morozov A.V., Denner J. (2016). New nested and real-time PCR systems for porcine cytomegalovirus (PCMV) detection and quantification. Arch. Virol..

[19] Widen B.F., Lowings J.P., Belak S., Banks M. (1999). Development of a PCR system for porcine cytomegalovirus detection and determination of the putative partial sequence of its DNA polymerase gene. Epidemiol. Infect..

[20] Hamel A.L., Lin L., Sachvie C., Grudeski E., Nayar G.P. (1999). PCR assay for detecting porcine cytomegalovirus. J. Clin. Microbiol..

[21] Plotzki E., Keller M., Ivanusic D., Denner J. (2016). A new Western blot assay for the detection of porcine cytomegalovirus (PCMV). J. Immunol. Methods.

[22] Liu X., Zhu L., Shi X., Xu Z., Mei M., Xu W., Zhou Y., Guo W., Wang X. (2012). Indirect-blocking ELISA for detecting antibodies against glycoprotein B (gB) of porcine cytomegalovirus (PCMV). J. Virol. Methods..

[23] Fiebig U., Holzer A., Ivanusic D., Plotzki P., Hengel H., Neipel F., Denner J. (2017). Antibody cross-reactivity between porcine cytomegalovirus (PCMV) and human herpesvirus-6 (HHV-6). Viruses.

[24] Denner J. (2015). Xenotransplantation and porcine cytomegalovirus. Xenotransplantation.

[25] Morozov V.A., Abicht J.M., Reichart B., Mayr T., Guethoff S., Denner J. (2016). Active replication of porcine cytomegalovirus (PCMV) following transplantation of a pig heart into a baboon despite undetected virus in the donor pig. Ann. Virol. Res..

[26] Lower R.R., Shumway N.E. (1960). Studies on the orthotopic homotransplantation of the canine heart. Surg. Forum..

[27] Morozov V.A., Ludwig S., Ludwig B., Rotem A., Barkai U., Bornstein S., Denner J. (2016). Islet Cell Transplantation from Göttingen Minipigs to Cynomolgus Monkeys, Analysis of Virus Safety. Xenotransplantation.

[28] Morozov V.A., Plotzki E., Rotem A., Barkai U., Denner J. (2016). Extended Microbiological Characterization of Göttingen Minipigs, Porcine Cytomegalovirus and Other Viruses. Xenotransplantation.

[29] Morozov V.A., Morozov A.V., Rotem A., Barkai U., Bornstein S., Denner J. (2015). Extended Microbiological Characterization of Göttingen Minipigs in the Context of Xenotransplantation, Detection and Vertical Transmission of Hepatitis E Virus. PLoS ONE.

[30] Abicht J.M., Mayr T.A., Reichart B., Plotzki E., Güthoff S., Falkenau A., Kind A., Denner J. (2016). Hepatic Failure After Pig Heart Transplantation Into a Baboon, No Involvement of Porcine Hepatitis E Virus. Ann. Transplant..

[31] Mueller N.J., Barth R.N., Yamamoto S., Kitamura H., Patience C., Yamada K., Cooper D.K., Sachs D.H., Kaur A. (2002). Activation of cytomegalovirus in pig-to-primate organ xenotransplantation. J. Virol..

[32] Ehlers B. (2002).

[33] Chmielewicz B., Goltz M., Franz T., Bauer C., Brema S., Ellerbrok H., Beckmann S., Rziha H.J., Lahrmann K.H., Romero C. (2003). A novel porcine gammaherpesvirus. Virology.

[34] Duvigneau J.C., Hartl R.T., Groiss S., Gemeiner M. (2005). Quantitative simultaneous multiplex real-time PCR for the detection of porcine cytokines. J. Immunol. Methods.

[35] Jothikumar N., Cromeans T.L., Robertson B.H., Meng X.J., Hill V.R. (2006). A broadly reactive one-step real-time RT-PCR assay for rapid and sensitive detection of hepatitis E virus. J. Virol. Methods.

[36] Mueller N.J., Livingston C., Knosalla C., Barth R.N., Yamamoto S., Gollackner B., Dor F.J., Buhler L., Sachs D.H., Yamada K. (2004). Activation of porcine cytomegalovirus, but not porcine lymphotropic herpesvirus, in pig-to-baboon xenotransplantation. J. Infect. Dis..

[37] Gu W., Zeng N., Zhou L., Ge X., Guo X., Yang H. (2014). Genomic organization and molecular characterization of porcine cytomegalovirus. Virology.

[38] Grote A., Hiller K., Scheer M., Münch R., Nörtemann B., Hempel D.C., Jahn D. (2005). JCat: A novel tool to adapt codon usage of a target gene to its potential expression host. Nucl. Acids Res..

[39] Plotzki E., Keller M., Ehlers B., Denner J. (2016). Immunological Methods for the Detection of Porcine Lymphotropic Herpesviruses (PLHV). J. Virol. Methods.

[40] Sekiguchi M., Shibahara T., Miyazaki A., Tajima T., Shimizu S., Kabali E., Takano Y., Sasaki Y., Kubo M. (2012). In situ hybridization and immunohistochemistry for the detection of porcine cytomegalovirus. J. Virol. Methods.

[41] Hall T.A. (1999). BioEdit, a user-friendly biological sequence alignment editor and analysis program for Windows 95/98/NT. Nucl. Acids. Symp. Ser..

[42] Fiebig U., Runge C., Denner J. (2017).

[43] Shibahara T. (2017).

[44] Morozov V.A., Heinrichs G., Denner J. (2017). Effective Detection of Porcine Cytomegalovirus Using Non-Invasively Taken Samples from Piglets. Viruses.

[45] Neumann E.J., Ramirez A., Schwartz K.J. (2010). Swine Disease Manual.

[46] Guedes M.I.M.C., Risdah I.J.M., Wiseman B., Molitor T.W. (2004). Reactivation of porcine cytomegalovirus through allogenic stimulation. J. Clin. Microbiol..

[47] Davison A.J. (2010). Herpesvirus systematics. Vet. Microbiol..

[48] Jurak I., Brune W. (2006). Induction of apoptosis limits cytomegalovirus cross-species infection. EMBO J..

[49] Whitteker J.L., Dudani A.K., Tackaberry E.S. (2008). Human fibroblasts are permissive for porcine cytomegalovirus in vitro. Transplantation.

[50] Blewett E., White G., Saliki J., Eberle R. (2001). Isolation and characterization of an endogenous cytomegalovirus (BaCMV) from baboons. Arch. Virol..

[51] Ross T.G., Rogers R.P., Elfrink N., Bray N., Blewett E.L. (2005). Detection of baboon cytomegalovirus (BaCMV) by PCR using primers directed against the glycoprotein B gene. J. Virol. Methods.

[52] Michaels M.G., Jenkins F.J., St Geroge K., Nalesnik M.A., Starzl T.E., Rinaldo C.R. (2001). Detection of Infectious Baboon Cytomegalovirus after Baboon-to-Human Liver Xenotransplantation. J. Virol..

[53] Michaels M.G., Alcendor D., St George K., Rinaldo C.R., Ehrlich G.D., Becich M.J., Hayward G.S. (1997). Distinguishing baboon cytomegalovirus from human cytomegalovirus, Importance for xenotransplantation. J. Infect. Dis..

