# Supplementary material for: Distribution of Porcine Cytomegalovirus in Infected Donor Pigs and in Baboon Recipients of Pig Heart Transplantation
Source: Viruses. 2018 Feb 6;10(2):66. doi: 10.3390/v10020066 (PMC5850373; doi:10.3390/v10020066)
Supplement: Supplementary file 1 [file viruses-10-00066-s001.pdf]

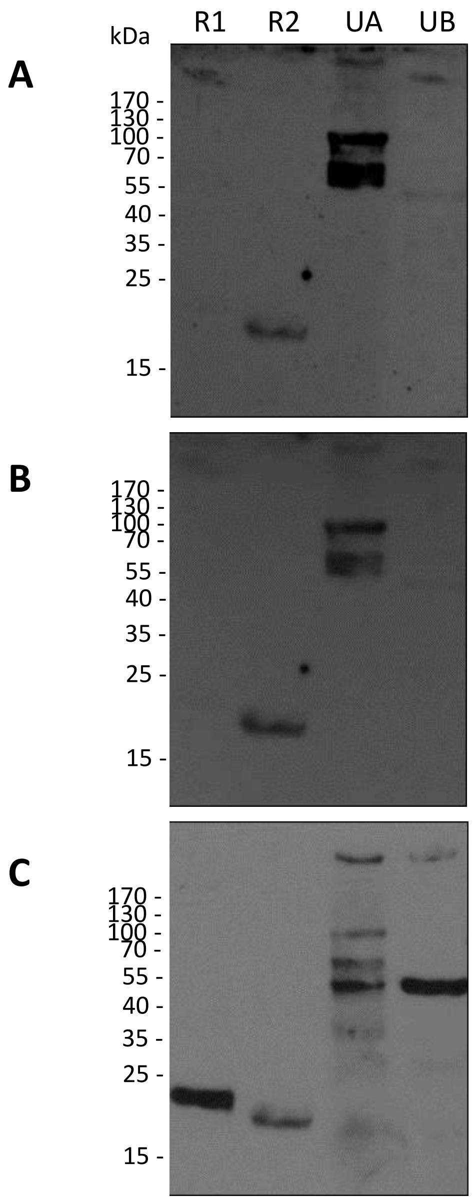

A
B
C
kDa
R1
R2
UA
UB
170
130
100
70
55
40
35
25
15

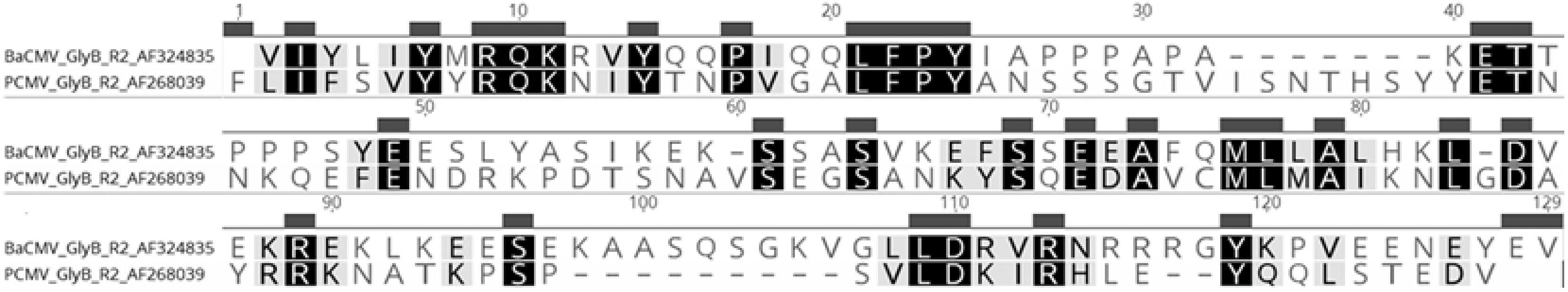
1
10
20
30
40
BaCMV_GlyB_R2_AF324835 VIYLIYMRQKRVYQQPIQQLFPYIAPPPAPA-------KETT
PCMV_GlyB_R2_AF268039 FLIFSVYYRQKNIYTNPVGALFPYANSSSGTVISNTHSYYETN
50
60
70
80
BaCMV_GlyB_R2_AF324835 PPPSYEESLYASIKEK-SSASVKEFSSEEAFQMLLALHKL-DV
PCMV_GlyB_R2_AF268039 NKQEFENDRKPDTSNAVSEGSANKYSQEDAVCMLMAIKNLGDA
90
100
110
120
129
BaCMV_GlyB_R2_AF324835 EKREKLKEESEKAASQSGKVGLLDRVRNRRRGYKPVEENEYEV
PCMV_GlyB_R2_AF268039 YRRKNATKPSP----------SVLDKIRHLE--YQQLSTEDV
